# Inflammation-linked aging signals in frozen single-cell foundation models: donor-aware detection and robustness testing

**DOI:** 10.1007/s10522-026-10471-8

**Published:** 2026-07-28

**Authors:** Ihor Kendiukhov

**Affiliations:** Department of Computer Science, University of Tübingen, Tübingen, Germany

**Keywords:** Aging, Inflammaging, Single-cell transcriptomics, Foundation models, Mechanistic interpretability, Sparse autoencoders, Immune aging, Confound control

## Abstract

Single-cell foundation models such as scGPT and Geneformer are large neural networks trained on human single-cell RNA-seq data. They were never shown chronological age during training. Do their internal representations nevertheless encode aging biology in a way that can be interpreted, and how should we test whether an apparent aging signal is real biology rather than an artifact of which donors and cell types happened to be sampled?. We applied a nine-step evaluation pipeline to two foundation models (frozen, no fine-tuning) and five PBMC datasets containing 4 to 5 million cells from ∼2,000 donors with chronological age. Each step is one specific test: can we read age out of the model’s representation; does the representation place age along a clean axis; do sparse-feature decompositions surface aging-related programs; do the two models agree at the pathway level; do targeted perturbations of those features change predicted age in the expected direction; and finally, does the signal survive when we resample cells so that young and old donors have matched cell-type composition (removing the most obvious confound). (1) The foundation models encode age but do not predict it better than a 50-component PCA of gene expression: in all five cohorts the PCA baseline matches or exceeds the best foundation-model probe. What they add is a complementary interpretability mode—sparse-feature decomposition and activation-level intervention—rather than predictive power; a PCA of gene expression is itself interpretable through its loadings, so the contribution here is the evaluation framework that adjudicates such signals, not a claim that foundation models predict age better. Randomly reinitialising Geneformer’s weights destroys most of its age signal (-0.107 balanced-accuracy points), while doing the same to scGPT’s layer 9 changes essentially nothing—so the two models encode age asymmetrically. (2) Sparse autoencoders surface 132 robust aging-related features across the two models, of which 193 cross-model pairs match each other at pathway level, concentrated in inflammation. The shared inflammation signal resolves into specific submodules: TNF / NF-κB classical and type-II IFN-γ (both models agree), complement (scGPT-specific). (3) The strongest aging signal is Geneformer’s NF-κB program in the AIDA phase 1 v2 cohort. Pushing those features in the “older” direction increases predicted age by 0.15 expected-age units; pushing them the opposite way decreases it; pushing along random unrelated directions does neither—a three-way directional check we call the “strict gate”. When cells are resampled so that the age groups have matched cell-type composition (the strictest control), the directional effect shrinks ∼3× but *7 of 8 resampling seeds still pass* the strict gate. One in eight resamplings fully nullifies the effect. The directional aging signal therefore survives confounder removal on most realizations, at attenuated magnitude. An external check on the Yazar OneK1K cohort (981 donors, fully separate from AIDA) reproduces the workflow on a known-strong biological axis (sex), with results within 10% of the AIDA contrast—evidence that the test is calibrated and transfers off-cohort. The paper’s primary contribution is an evaluation framework for deciding when an apparent aging signal in a single-cell foundation model is biology rather than sampling structure. Applied here, it shows that frozen foundation models carry a recoverable aging signal concentrated in NF-κB and IFN-γ inflammation submodules—biology that is already established at the gene-expression level, recovered zero-shot from models never trained on age. Reporting both an unrestricted contrast and a composition-matched contrast as side-by-side specificity tests—not just the headline number—is the framework’s central recommendation.

## Introduction

Aging is a systems-level process with hallmarks in chronic inflammation, senescence-associated secretory signaling, stem-cell dysfunction, and altered intercellular communication (Lopez-Otin et al. 2013, 2023; Kennedy et al. 2014; Campisi et al. 2019). Inflammaging is well established at the gene-expression level: persistent innate-immune activation, NF-κB signaling, senescence-associated secretory phenotypes, and adaptive-immunity rewiring all track chronological age (Franceschi et al. 2000; Franceschi and Campisi 2014; Furman et al. 2019; Ferrucci and Fabbri 2018; Nikolich-Zugich 2018; Goronzy and Weyand 2019; Salminen et al. 2008; Chien et al. 2011; Hernandez-Segura et al. 2018; Basisty et al. 2020; Zhang et al. 2013). DNA-methylation clocks, blood transcriptomic signatures, and large atlas-scale references can decode age from molecular profiles (Horvath 2013; Ake et al. 2019a; Marjolein et al. 2015; Almanzar et al. 2020; Schaum et al. 2020; The Tabula Sapiens Consortium et al. 2022; Yazar et al. 2022).

We are explicit about what is and is not new here. The biology we recover—that aging is inflammatory, organised around NF-κB and IFN-γ programs—is already established at the gene-expression level (references above); we do not claim a new aging mechanism. The contribution is methodological and representational, in three parts, the first of which is primary:

1. *An evaluation framework that adjudicates apparent aging signal (primary contribution): *Apparent aging signal in scFMs is easy to detect, but separating biology from donor identity, cell-type composition, and cohort-specific imbalance is hard. We treat that separation as the unit of evidence and build a nine-block pipeline whose final two blocks—a fully composition-matched forward-pass rerun and a multi-seed matched panel—are decisive specificity tests. The strongest claim in our analysis attenuates substantially under those tests, and the framework’s value is precisely that it quantifies how much. The framework is representation-agnostic: it applies equally to PCA factors, gene-set scores, or foundation-model features.
2. *Mechanistic-interpretability tooling applied to aging in frozen scFMs: *We apply tooling developed for circuits and sparse features in large neural networks (Olah et al. 2020; Bau et al. 2017; Bricken et al. 2023; Templeton et al. 2024) to single-cell foundation models (Theodoris et al. 2023; Cui et al. 2024; Vaswani et al. 2017) on aging data. Prior aging+scRNA work uses prediction or differential expression. We instead resolve the latent space into sparse features, test cross-model agreement at the pathway level, and run targeted activation-space interventions with donor-bootstrap CIs.
3. *Zero-shot recoverability from non-aging-trained models*: Neither scGPT nor Geneformer received chronological age labels during pretraining; their training objectives (gene-expression reconstruction; gene-rank prediction) are aging-agnostic. Recovering a cross-model-convergent inflammatory program from these frozen embeddings is evidence that this known aging biology is implicit in the gene-expression statistics the models were trained on—a statement about the representations, not a new biological finding.

### Why use frozen foundation models, given that simpler baselines predict equally well?

A 50-component PCA on gene expression, fit on the same donor-aware splits, matches or beats the best frozen foundation model on age prediction across all five cohorts (Section 3.1). On prediction alone, foundation models are not winning. They are also not the only interpretable option: a PCA-plus-regression model is itself interpretable, since each principal component is a linear combination of genes, so the genes and pathways driving an age prediction can be read off the loadings directly and tested by gene-set enrichment. We do not claim foundation models are uniquely or even more interpretable. What they offer is a *different* mode of interpretability: their representations admit sparse, more nearly monosemantic feature decomposition, can be aligned across two architecturally distinct models at the pathway level, and—because the model is a forward-pass generator—can be perturbed at the activation level to test whether pushing a feature changes the predicted age. These capabilities are complementary to, not a replacement for, loading-based enrichment of a linear model. The rest of the paper exploits this mode while reporting the linear baselines alongside, and the decisive specificity tests (Blocks 7–9) apply to any of these representations.

## Methods

### Pipeline overview

The pipeline answers four questions in order, each more demanding than the last.

1. *Is age in the model’s representation at all? (Blocks 1–2.)*: We train a simple classifier on the model’s per-cell embedding to predict age class, with a strict rule that no donor’s cells appear in both training and test (“donor-aware splits”). We then check whether age sits on a stable global axis in the embedding by measuring three geometric statistics. The probe almost always succeeds; the geometry test almost always fails—meaning age is detectable but not arranged along a single global axis, so we need a sparse decomposition to find it.
2. *Which specific features of the model encode age, and do two different models agree?*: (Blocks 3–4.) We fit sparse autoencoders on the per-cell embeddings; each autoencoder feature is then scored against age, donor, and cell type. Only features that vary with age more than they vary with donor identity are retained. We then check, for each retained feature, which curated biological pathway its top genes overlap with (e.g. NF-κB, type-I interferon, complement), and look for cases where scGPT and Geneformer point at the same pathway in the same dataset.
3. *Does perturbing those features actually change predicted age?: *(Blocks 5–6.) For each candidate aging feature, we push its activation in the “older” direction, in the “younger” direction, and along an unrelated random direction, then ask the probe to predict age on the perturbed embeddings. If the older-direction push raises the prediction, the younger-direction push lowers it, and the random push does neither—with confidence intervals from donor-level resampling—we count this as a directional intervention. The combined test is what we call the “strict gate”.
4. *Does the signal survive confound removal? (Blocks 7–9.): *The most likely source of false positives in single-cell aging analyses is cell-type composition: older donors have more of some cell types and fewer of others, and a model can learn to predict “age” by simply detecting that mix. We address this two ways. First, we reweight donor-level statistics so the age groups have matched compositions (a soft control). Second, and decisively, we resample cells per donor so that age groups have matched cell-type fractions *before* the model’s forward pass—removing the confound by construction—and rerun the entire pipeline (a hard control). We do this for eight different resampling seeds (Block 9) to test how much the verdict depends on the particular resampling.

**Fig. 1:**
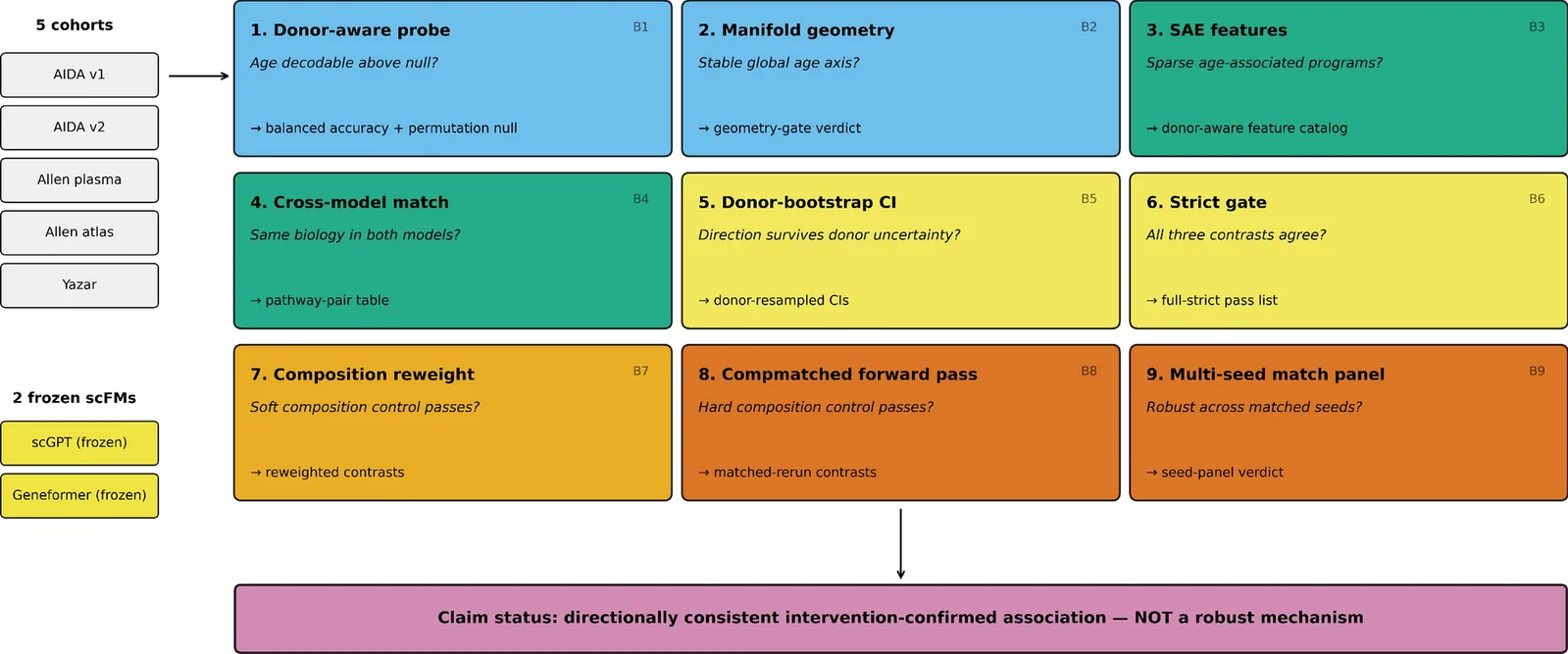
Donor-aware mechanistic-interpretability pipeline. Five age-labeled cohorts and two frozen scFMs feed a nine-block evaluation. Blocks 1–4 establish a candidate signal; 5–6 qualify it as directional; 7–9 stress-test it against the composition confound. Promoted claims are the conjunction of all nine outcomes

Figure 1 shows the same pipeline visually.

### Datasets

We work with five age-labeled human immune cohorts. Table 1 reports cell counts, donor counts, age distributions, sex and ethnicity composition, and which sub-analysis each cohort feeds.

**Table 1:** Cohorts used in this study. All five are PBMC-derived. Full-cohort statistics; the sampled-analysis subset is 3000 cells per cohort with donor-aware splits

**Cohort**	**Cells**	**Donors**	**Age med./IQR**	**Sex F/M**	**Ethnicity (top groups)**
AIDA phase 1 v1	1,058,909	508	41/33–51	56/44	Korean, Japanese, SG-Chinese/Indian/Malay
AIDA phase 1 v2	1,265,624	625	40/31–49	56/44	Korean, Japanese, SG-CMI, Thai, Indian
Allen aging plasma	455,893	234	59/50–75	59/41	Caucasian, Asian, other
Allen immune atlas	1,821,725	108	34/28–58	55/45	Caucasian (89%), Asian, other
Yazar donor cohort	1,248,980	981	67/56–76	58/42	Australian (100%)

All five are PBMC-derived. Full-cohort statistics; the sampled-analysis subset is 3000 cells per cohort with donor-aware splits

The two AIDA cohorts (Asian Immune Diversity Atlas, phase 1 v1 and v2) are the core deep-dive datasets because of their size and donor diversity. Allen immune atlas, Allen aging plasma cells, and the Yazar cohort (Yazar et al. 2022) provide external context. We treat external cohorts as stress-test contexts rather than interchangeable training pools because cross-cohort integration in single-cell data is sensitive to normalization, batch effects, donor structure, and pseudoreplication (Wolf et al. 2018; Hafemeister and Satija 2019; Stuart et al. 2019; Haghverdi et al. 2018; Korsunsky et al. 2019; Tran et al. 2020; Stephanie et al. 2018; Zimmerman et al. 2021; Squair et al. 2021).

### Models, baselines, and null calibrations

We use frozen **scGPT** (contextual layer scgpt_layer_09) (Cui et al. 2024) and frozen **Geneformer** (contextual representation) (Theodoris et al. 2023). Neither model is fine-tuned for age prediction.

For null calibration, we evaluate every age probe alongside three references: a 50-component PCA-on-expression baseline trained on the same donor-aware splits; a within-stratum permutation null at $n_{\textrm{perm}}=25$; and the trivial 1/*K* chance balanced accuracy. Section 3.1 reports all three and the foundation-model probes side-by-side.

### Interpretability blocks

**Block 1—Age probe.** *Can we read age out of the model’s per-cell embedding?* For each cohort and model, we train a logistic-regression classifier (with L2 regularisation) to predict the cell’s age class from its embedding. Splits are donor-aware: every donor’s cells go entirely to the train set or entirely to the test set, never both, so the probe cannot succeed by memorising individual donors (Efron 1979). Targets are four age classes (quartile bins).

**Block 2—Manifold geometry.** *If age is encoded, is it arranged along a clean global axis?* For each cohort and representation we compute three geometric statistics on the embedding’s PCA spectrum: the participation ratio (how many directions carry the variance), the maximum absolute correlation between age and any top principal axis, and a per-class silhouette score for age. A dataset “passes the geometry gate” only when all three jointly indicate a stable global age axis. Most cohorts fail this gate, which is why we then turn to sparse decomposition.

**Block 3—Sparse autoencoders (SAEs).** *Which specific features of the embedding encode age, and are those features clean?* We train sparse overcomplete autoencoders on the per-cell embeddings, following the dictionary-learning literature (Bricken et al. 2023; Templeton et al. 2024). Each learned feature is then scored against three labels: age, donor, and cell type. We retain only features whose age effect (as measured by $\eta ^2$, the variance explained by age) is at least as large as its donor effect, that pass a donor-permutation test, and that are stable across multiple SAE training seeds. We call these *donor-aware robust features*.

**Block 4—Cross-model pathway matching.** *Do scGPT and Geneformer agree on the biology?* For each robust feature, we look at the genes whose expression correlates most strongly with that feature, and intersect the top-50 gene list against curated pathway gene sets covering inflammation/NF-κB, type-I antiviral interferon, type-II IFN-γ, IL-6/JAK-STAT, NLRP3 inflammasome, complement, proteostasis/UPR, and senescence/SASP. We assign each feature its strongest-overlap pathway with hypergeometric significance. A *cross-model pair* is one scGPT feature and one Geneformer feature in the same cohort, with the same primary pathway and the same direction of correlation with age.

**Block 5—Targeted perturbations of aging features.** *If we push these features harder in the “older” direction, does the model think the cell is older?* For each candidate pathway we push its SAE features in three directions: *aging push*, *young push* (the opposite sign), and a magnitude-matched *random push* along unrelated directions. We then ask the probe to predict age on the perturbed embedding. To isolate the direction-specific effect from the autoencoder’s encode-decode reconstruction noise (which would otherwise dominate when the SAE was trained on a different forward pass than the one used at evaluation), we evaluate the perturbation as a hidden-space delta:

$$\begin{aligned} \Delta _{\text {predicted age}}&= \textrm{probe}(X + \Delta _{\text {push}}) - \textrm{probe}(X), \\ \Delta _{\text {push}}&= \textrm{decode}\bigl (\textrm{encode}(X) + \textrm{push}\bigr ) - \textrm{decode}\bigl (\textrm{encode}(X)\bigr ). \end{aligned}$$

We aggregate to donor-level means and obtain 95% confidence intervals by non-parametric resampling of donors (not cells) (Efron 1979).

**Block 6—Strict gate.** *Does the perturbation pass a three-way directional check?* We promote a candidate aging feature only if all three perturbations behave as expected: the aging push raises predicted age, the young push lowers it, and the random push does neither—and the confidence intervals on all three contrasts exclude zero. We refer to this combined test as the *strict gate*. Section 3.7 reports an empirical calibration of how strict the gate actually is.

**Blocks 7–9—Confound controls.** *Is the apparent aging signal driven by cell-type composition rather than aging biology?* Older and younger donors have different mixes of immune cell types; a model can learn to predict “age” by detecting the mix. We address this in three increasingly strict ways. Block 7 reweights donor-level statistics so that age groups have matched cell-type compositions on average (a soft control). Block 8 actually resamples cells per donor so that age groups have identical cell-type fractions *before* the foundation model’s forward pass—removing the composition confound by construction (a hard control). Block 9 runs Block 8 with eight different random seeds for the resampling, so the verdict does not depend on any single sample realisation.

### Glossary

**Figure:**
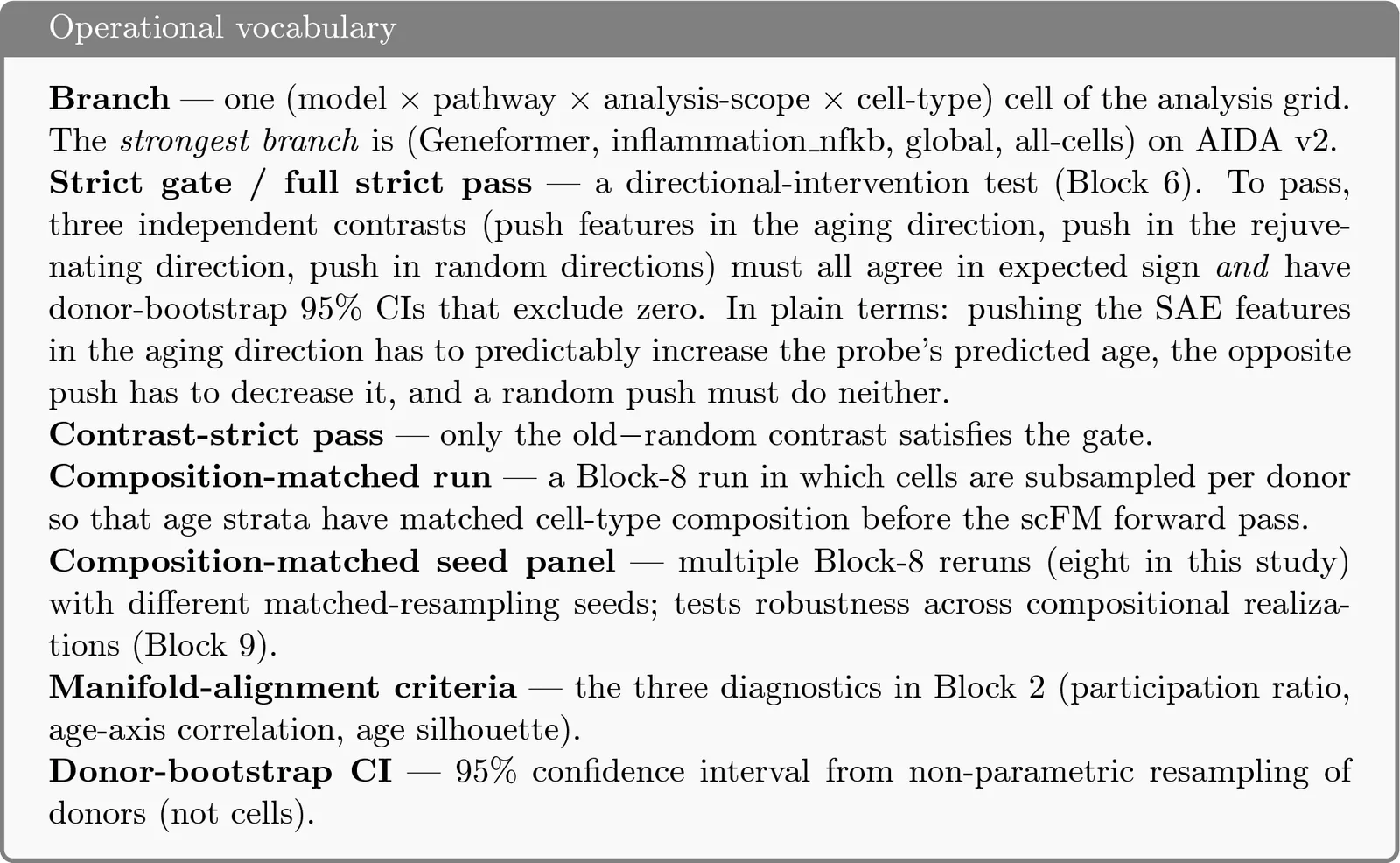


### Statistical safeguards

Single-cell analyses are uniquely vulnerable to optimistic results when cells are treated as independent evidence, when donor effects leak across splits, or when batch correction silently redefines the contrast (Crowell et al. 2020; Zimmerman et al. 2021; Squair et al. 2021). We use donor-aware partitioning, donor-bootstrap CIs, and contrast checks that survive composition reweighting and full composition-matching.

### Exploratory methylation extension

We adapted the same interpretability logic to MethylGPT (Ying et al. 2024) on the AltumAge pan-tissue methylation compendium (Paulo De Lima et al. 2022). The MethylGPT checkpoint expects 49,156 CpG tokens; faithful full-length inference was not feasible on available hardware, so we used an explicitly approximate 512-CpG window procedure (with shifted, random, and sparse-selection layouts) and annotated overlap against established methylation clocks (Horvath 2013; Horvath et al. 2018; Levine et al. 2018; McEwen et al. 2020; Ake et al. 2019b; Belsky et al. 2022). We treat this branch as cross-modality triangulation, not direct replication.

## Results

### Foundation models detect age, but not better than a gene-expression PCA baseline

**Fig. 2:**
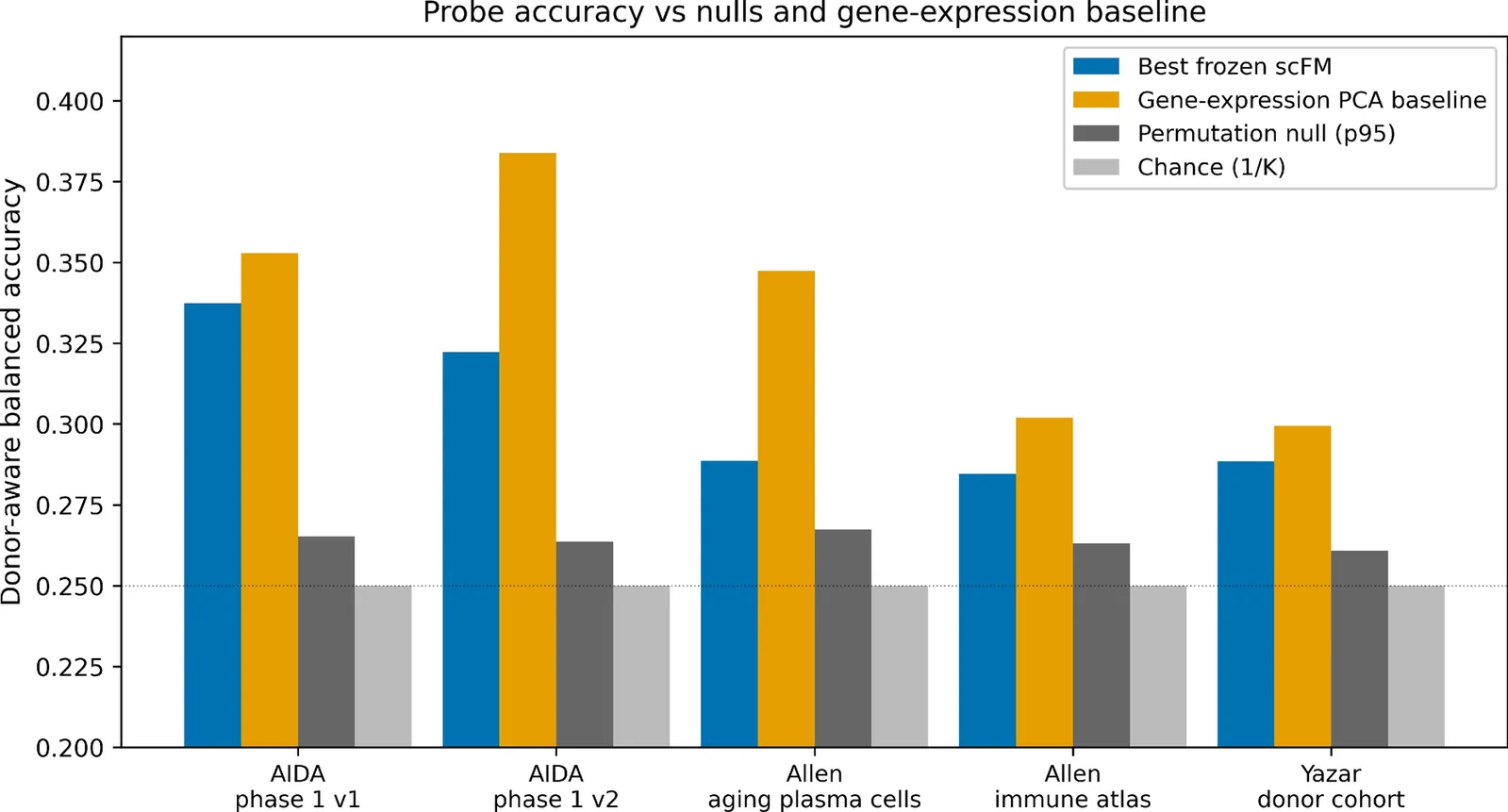
Probe accuracy across cohorts vs. nulls and the gene-expression PCA baseline. Frozen scFMs encode genuine age signal (above permutation null) but do not exceed the simpler PCA-on-expression baseline. The pretraining advantage is interpretive, not predictive; this framing organises the analyses that follow

Both foundation models contain real age signal: in every cohort the probe trained on their embeddings beats both chance (1/K=0.25 for K=4 age classes) and the 95th percentile of a within-stratum permutation null. The fairer comparison, however, is against a much simpler reference: a 50-component PCA of gene expression, fed to the same logistic-regression probe on the same donor-aware splits. Table 2 shows that in 5 of 5 cohorts the PCA baseline matches or exceeds the best foundation-model probe. On AIDA phase 1 v2 the gene-expression PCA reaches 0.384 donor-aware balanced accuracy, while the best foundation model (Geneformer) reaches only 0.322. Figure 2 visualises this directly. *The foundation models are not better at predicting age than a PCA of the underlying gene expression.* They are studied in the rest of the paper not as predictors but as objects whose internal structure supports the complementary interpretability mode described in Section 2.4.

**Table 2:** Null-calibrated probe performance. Best frozen scFM, gene-expression PCA baseline, permutation-null mean and 95th percentile, and chance (1/*K*). All probes use the same donor-aware splits.

**Cohort**	**Donors**	**scFM BA ± SD**	**Gene-expr BA**	**Perm null (p95)**	**Chance**	**scFM − expr**
AIDA v1	504	0.337 ± 0.013	0.353	0.249 (0.265)	0.250	-0.016
AIDA v2	622	0.322 ± 0.008	0.384	0.251 (0.264)	0.250	-0.062
Allen plasma	225	0.289 ± 0.015	0.347	0.250 (0.267)	0.250	-0.059
Allen atlas	108	0.285 ± 0.007	0.302	0.248 (0.263)	0.250	-0.017
Yazar	940	0.288 ± 0.007	0.299	0.248 (0.261)	0.250	-0.011

Best frozen scFM, gene-expression PCA baseline, permutation-null mean and 95th percentile, and chance (1/K). All probes use the same donor-aware splits.

### Age is detectable but not arranged along a clean global axis

None of the five cohorts pass the geometry test (Block 2): the participation ratio, age–axis correlation, and age silhouette score do not jointly indicate a stable global age axis in the embedding space. In other words, age is decodable, but it is not stored along a single dominant direction. The signal must therefore live in sparser, possibly cell-type-local, possibly non-axis-aligned combinations of features—which is the regime where sparse-decomposition tools become useful.

### Sparse autoencoders find 132 robust aging-related features

**Fig. 3:**
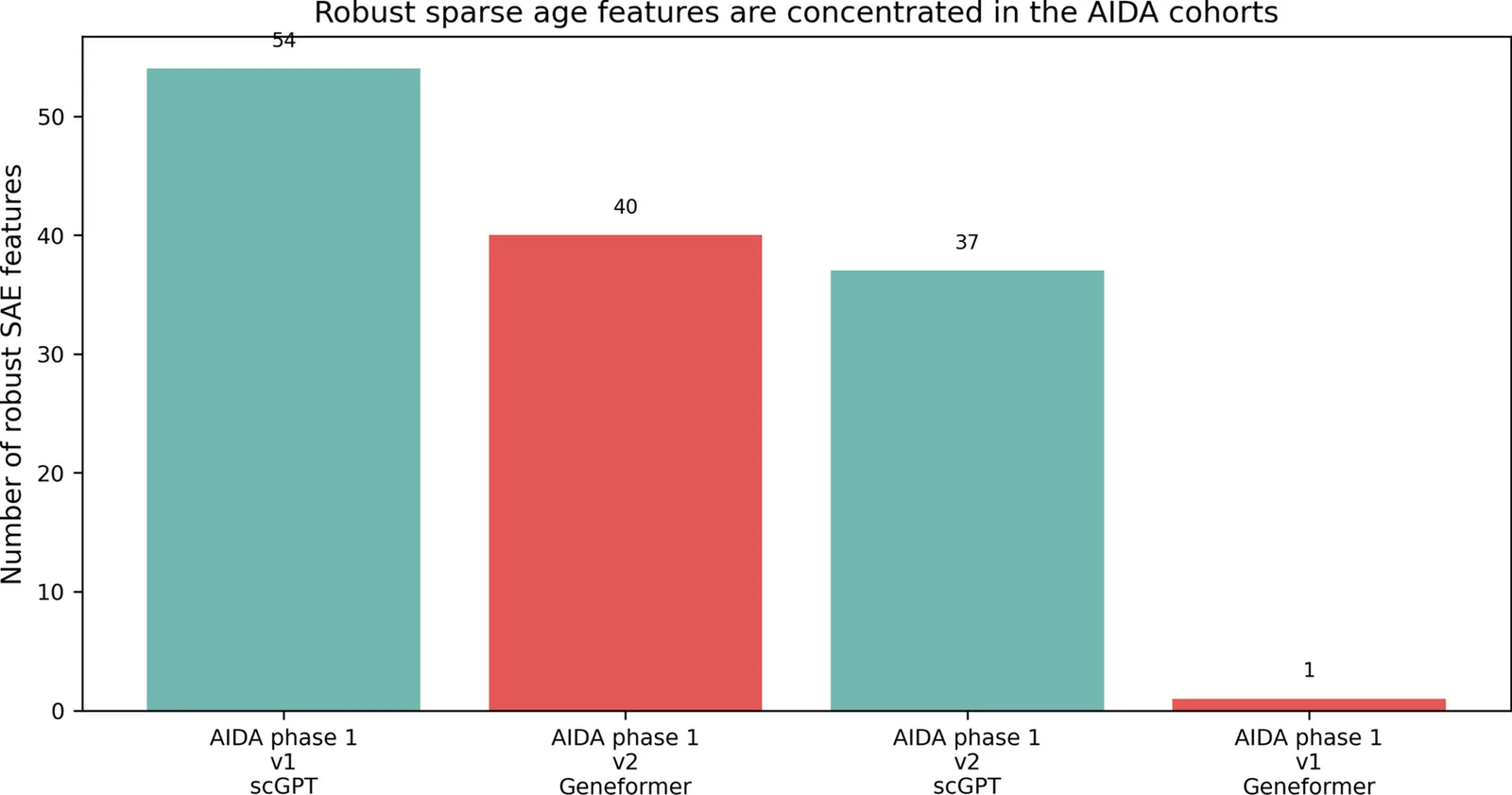
Donor-aware robust SAE features by cohort and model. The signal is concentrated in the AIDA cohorts

Of all features the sparse autoencoders learn, 132 pass our donor-aware robustness filter (91 in scGPT, 41 in Geneformer): they correlate more with age than with donor identity, survive a donor-permutation test, and replicate across SAE training seeds. Most of the signal comes from the two AIDA cohorts (Figure 3). When we then look for cases where one scGPT feature and one Geneformer feature share the same primary biological pathway and the same direction of age-correlation, we find 193 such cross-model pairs. Most of those pairs (175 in AIDA v2) sit in inflammation/NF-κB.

**Fig. 4:**
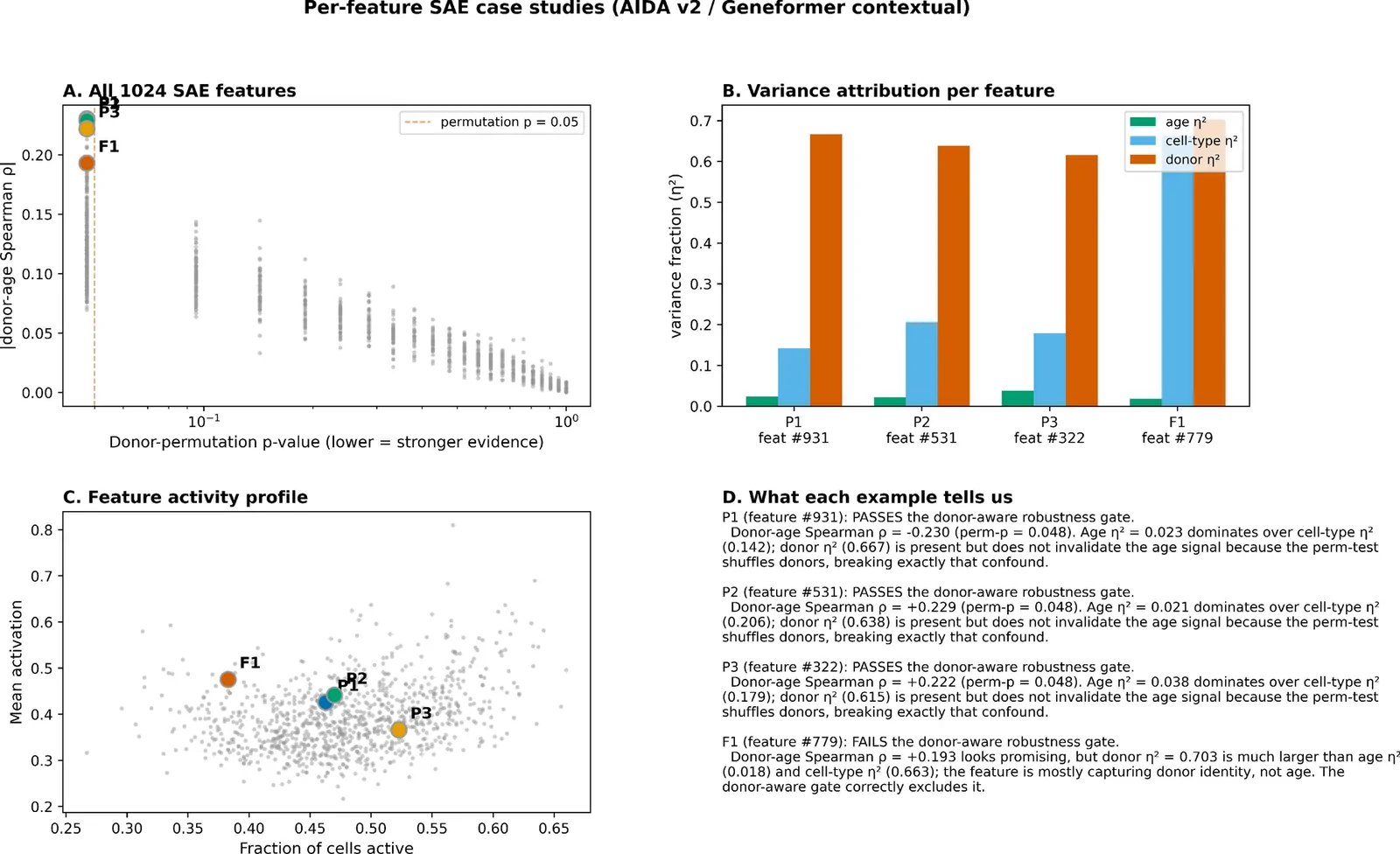
Per-feature SAE case studies (AIDA v2/Geneformer). Three passing features (P1–P3) and one failing feature (F1) shown across donor-age Spearman, variance attribution, and activity-profile panels. The failing feature has the highest donor $\eta ^2$ in the SAE; the donor-aware gate correctly excludes it

Figure 4 walks through four representative SAE features (three that pass donor-aware robustness, one that fails because donor $\eta ^2$ dominates over age $\eta ^2$). The figure shows what each panel of the per-feature analysis means, providing concrete examples that motivate the aggregate statistics elsewhere in this section.

### Inflammation breaks down into submodules; both models agree on TNF/NF-κB and IFN-γ but not on complement

Saying “aging features cluster in inflammation/NF-κB” is too coarse to be a useful biological finding—inflammation is a large family of related programs with very different upstream triggers and downstream effects. To distinguish them, we re-scored each of the 49 inflammation-NF-κB features against six curated submodule gene sets: TNF/NF-κB classical; type-I antiviral interferon; type-II IFN-γ; IL-6/JAK-STAT; NLRP3 inflammasome; and complement. Table 3 reports the resulting submodule assignments by cohort and model.

**Fig. 5:**
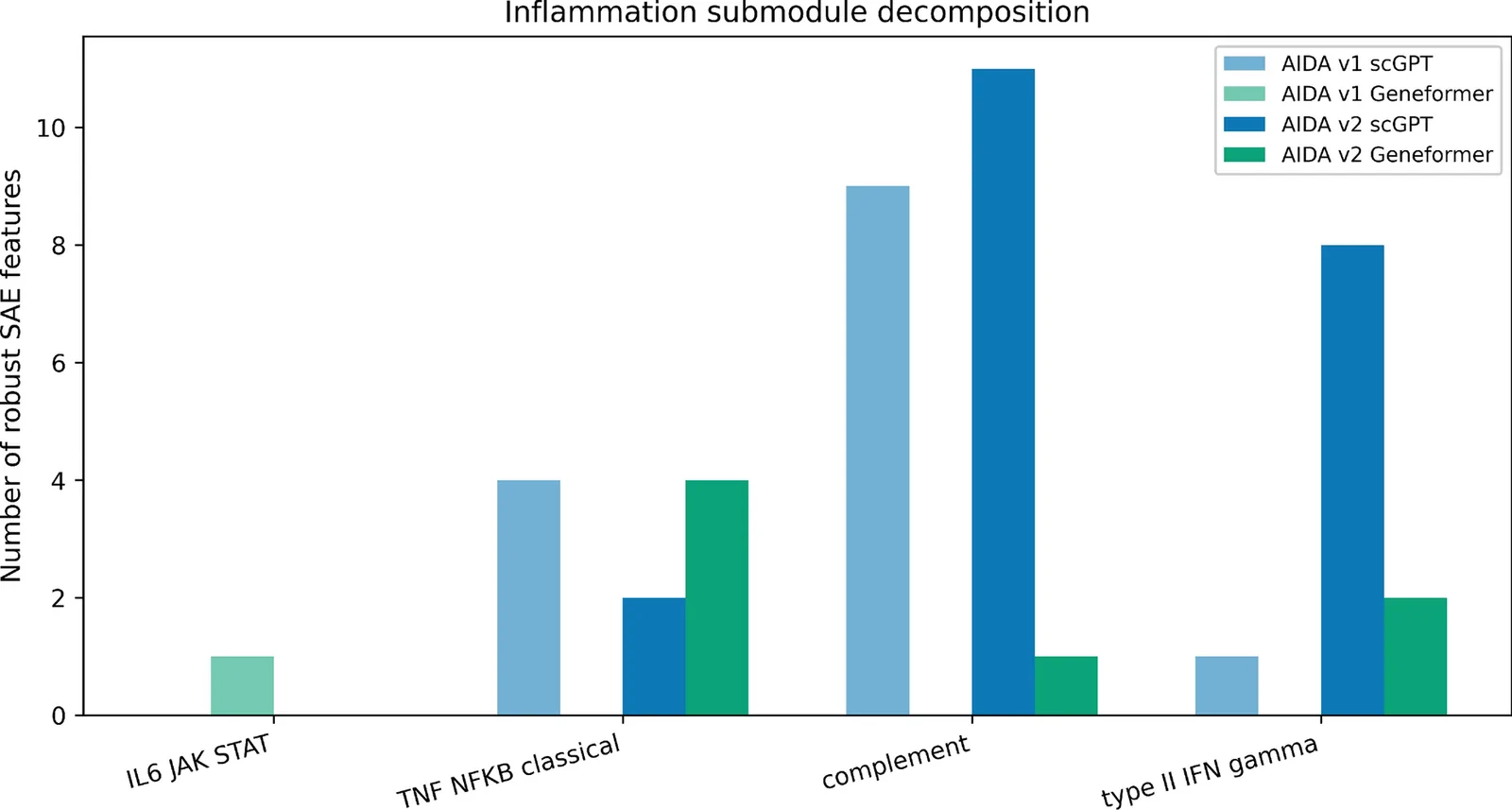
Resolved inflammation submodules across AIDA cohorts and models. Cross-model agreement concentrates in TNF/NF-κB classical and type-II IFN; complement is dominated by scGPT-specific features

Two patterns emerge. (i) In AIDA phase 1 v2, both scGPT and Geneformer converge on the same two submodules: TNF/NF-κB classical (2 cross-model pairs) and type-II IFN-γ (2 cross-model pairs). These are the canonical inflammaging axes; the agreement across two architecturally different foundation models is good evidence that they are recoverable from gene-expression statistics alone, regardless of pretraining objective. (ii) The complement submodule is dominated by scGPT-specific features (11 features in scGPT vs. 1 in Geneformer in AIDA v2). The two models therefore disagree on whether complement is part of the aging signature—a model-specific call rather than a cross-model robust finding (Fig. 5).

**Table 3:** Inflammation submodule features by cohort and model. Cross-model pair count = $\min (\text {scGPT features}, \text {Geneformer features})$ within submodule.

**Cohort**	**Submodule**	**scGPT feat.**	**Geneformer feat.**	**Cross-model pairs**
AIDA v1	IL-6/JAK-STAT	0	1	0
AIDA v1	TNF/NF-κB classical	4	0	0
AIDA v1	complement	9	0	0
AIDA v1	type-II IFN-γ	1	0	0
AIDA v2	TNF/NF-κB classical	2	4	2
AIDA v2	complement	11	1	1
AIDA v2	type-II IFN-γ	8	2	2

### Cell-type-specific monocyte signal: present in AIDA v1, absent in AIDA v2—and why

Restricting the analysis to CD14 monocytes alone gives a clean positive result on AIDA phase 1 v1: pushing inflammation/NF-κB features in the “older” direction increases predicted age by +0.036 in Geneformer (95% CI [+0.031,+0.040]) and +0.042 in scGPT (95% CI [+0.037,+0.047]). Both confidence intervals exclude zero. The same intervention on AIDA v2 monocytes produces uncertain or negative effects (Geneformer +0.002, CI [-0.0002,+0.004]; scGPT -0.001, CI [-0.002,-0.0004]).

**Fig. 6:**
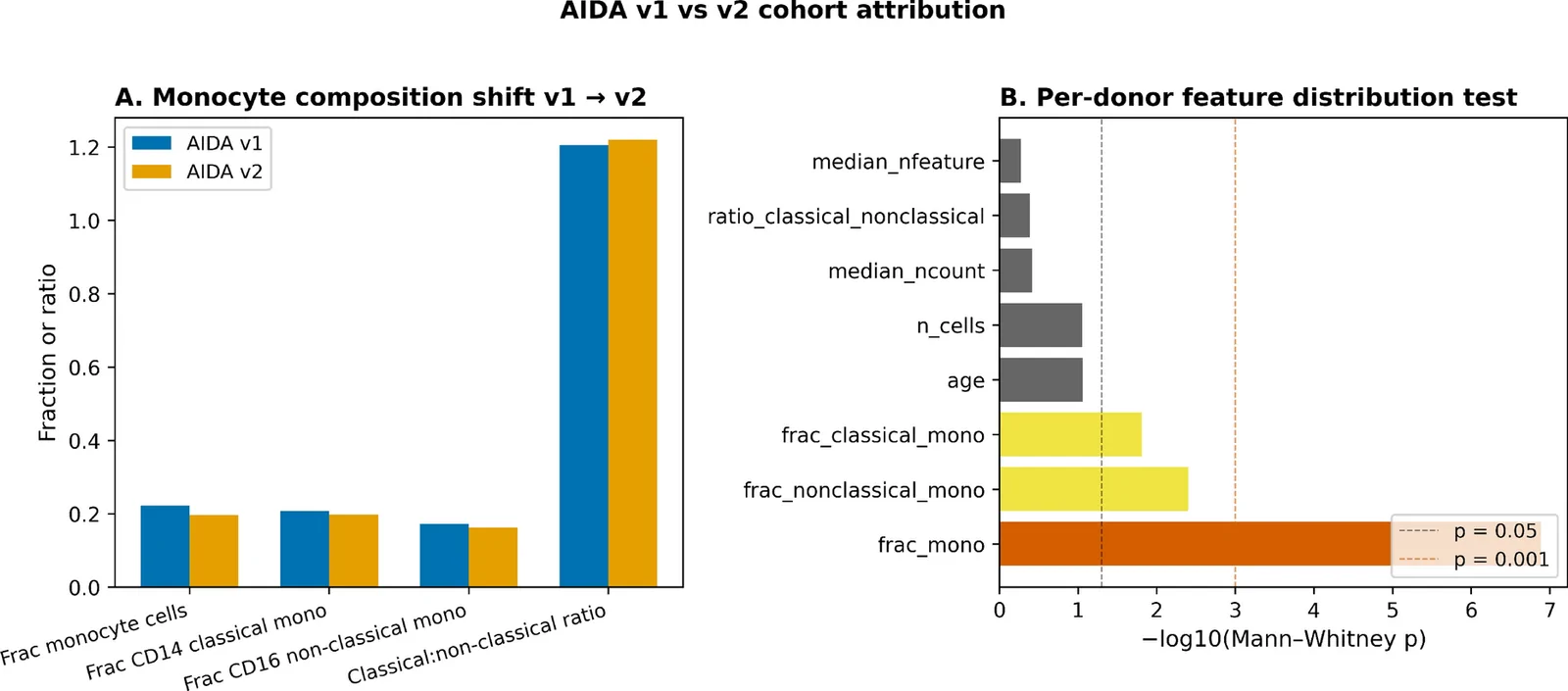
AIDA v1 vs v2 cohort attribution. (A) Monocyte composition shifts between cohorts. (B) Per-donor distribution tests; monocyte-fraction is the most strongly differing feature ($p=1.3\times 10^{-7}$)

The natural question is whether this v1→v2 drop reflects a real biological inconsistency in inflammaging, or some structural property of how the two cohorts were assembled. We compared the two cohorts directly (Figure 6) and found three structural differences that together explain the drop *without* requiring biology to change:

1. *Donor pool composition: *AIDA v2 has 117 more donors than v1, and adds two ethnicities not present in v1 (Thai and Indian). Aging-associated inflammation is partially shaped by genetic and environmental background; the broader v2 donor pool introduces between-donor heterogeneity that the donor-aware probe cannot fully average over.
2. *Monocyte fraction shift*: The per-donor fraction of monocytes drops from 0.21 in v1 to 0.19 in v2 (Mann–Whitney $p = 1.3\times 10^{-7}$). Since the inflammation contrast is driven specifically by classical CD14 monocytes, a population-level shift in their abundance attenuates the contrast at any fixed nominal age.
3. *Sequencing depth differences*: Median per-cell read counts and detected genes differ measurably between cohorts. Foundation-model embeddings depend on coverage (Geneformer uses gene-rank, scGPT uses expression magnitude), so a depth shift alters the activation profile of any fixed SAE feature.

The framework is doing what it should: a positive cell-type-restricted aging signal in a smaller, more homogeneous cohort can fail in a larger, more heterogeneous cohort even when the underlying biology is unchanged. The Allen-cohort replication of the monocyte intervention in §4.1 provides the third-cohort tiebreaker.

### The strongest aging signal: how it changes with sample size and under composition matching

*What we ask.* The strongest aging signal in the analysis is Geneformer’s NF-κB program in the AIDA phase 1 v2 cohort. Pushing those features in the “older” direction increases predicted age by +0.149 expected-age units (95% CI [+0.142,+0.156]). A natural worry is that any signal that weakens under stricter controls might simply be a sample-size artifact rather than a real biological attenuation. To tell those apart, we measured *how the effect changes as we vary the number of donors*.

*Empirical power curve.* We repeatedly subsampled the 424-donor pool at seven sample sizes between 50 and 400 donors (50 subsamples per setting, with 1000 donor-level bootstrap iterations per subsample), and ran the strict gate on each subsample. The baseline strict-gate pass rate was 1.00 at every donor count, with the contrast settling near +0.150 at every sample size—so the test is already saturated at n=50 donors, not power-limited.

**Fig. 7:**
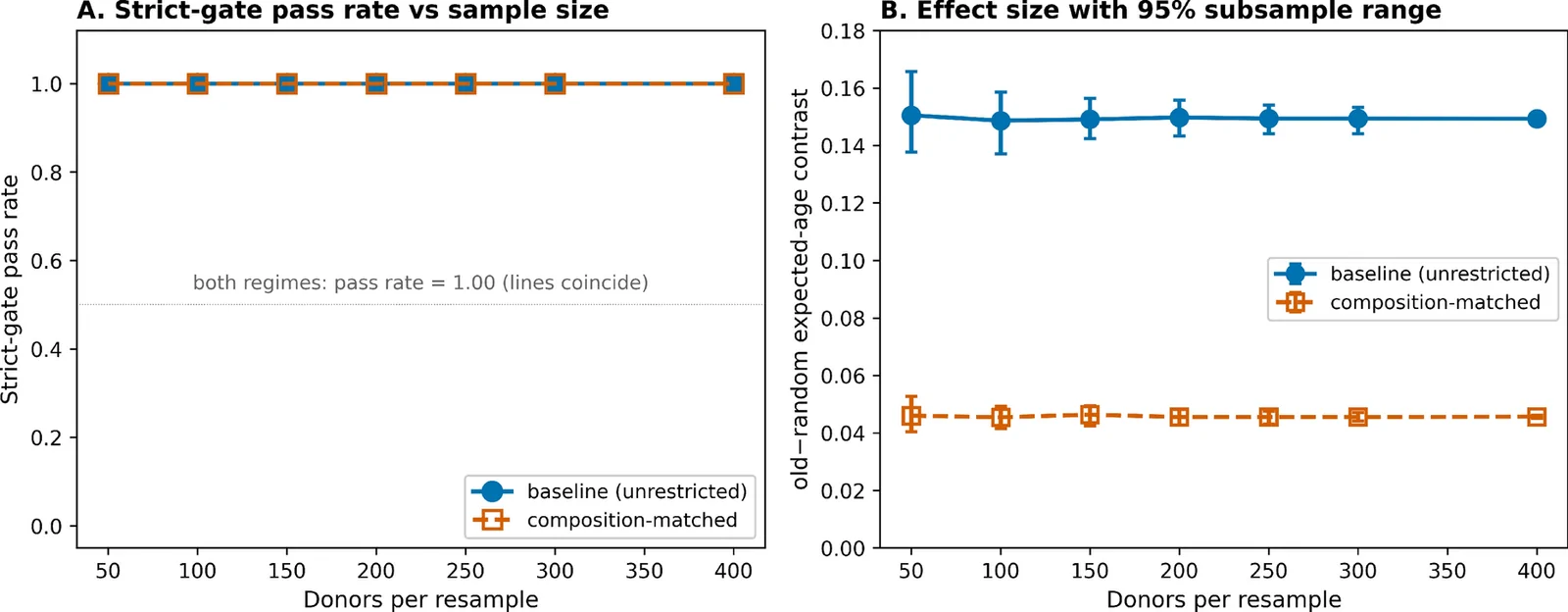
Empirical power curve. Left panel **A:** strict-gate pass rate versus number of donors per resample. Both the baseline (unrestricted, blue filled circles) and composition-matched (vermilion open squares) regimes reach a pass rate of 1.00 from n=50 onward; the two lines coincide at 1.00 and are drawn with distinct markers so neither is hidden. Right panel **B:** the old−random expected-age contrast with its 95% subsample range. The baseline contrast sits at ≈+0.150 across all sample sizes, while the composition-matched contrast saturates at ≈+0.046 ($\approx 3.2\times$ smaller). The signal is therefore not power-limited at either setting; composition matching attenuates the magnitude rather than the detectability

*Same procedure, but with composition matched.* We then repeated the same sample-size sweep on the composition-matched donor pool (cells subsampled per donor so that age groups have identical cell-type fractions; 8 matched-resampling seeds, 414 unique donors total). Strict-gate pass rate is again 1.00 across the entire range, but the contrast magnitude settles at +0.046 (95% CI [+0.045,+0.046]) at n=400—about 3.2× smaller than the unrestricted baseline contrast (Fig. 7).

*What this means.* Composition matching shrinks the aging contrast about three-fold, but does not erase it: sign and statistical significance both survive. The signal is therefore neither pure biology nor pure confound. The matched contrast (+0.046) is roughly 31% of the unrestricted contrast (+0.150), so cell-type composition accounts for about two-thirds of the unrestricted magnitude and roughly one-third survives the strictest control.

*Why match composition rather than regress it out?* A natural alternative is to fit a linear model that includes cell-type composition as covariates and ask how much of the age signal remains. Our composition-matched forward pass is a stronger, assumption-free version of exactly that control. Regressing out a composition term removes only the variance composition explains *linearly and additively* on the chosen scale; the resampling design instead equalises cell-type fractions across age strata *before* the foundation-model forward pass, so any composition contribution—linear or non-linear, and including interactions the model itself induces—is removed by construction. Because matching removes a superset of what a linear covariate adjustment removes, it is the more conservative estimate of the surviving fraction; a linear adjustment would, if anything, attribute *less* of the signal to composition and leave a larger residual. The ∼one-third figure should therefore be read as a lower bound on how much of the signal is composition-independent.

### How strict is the strict gate, in practice?

*What we ask.* The strict gate is meant to separate “real signal” from “signal driven by composition confounding”. Does it actually do that empirically?

**Fig. 8:**
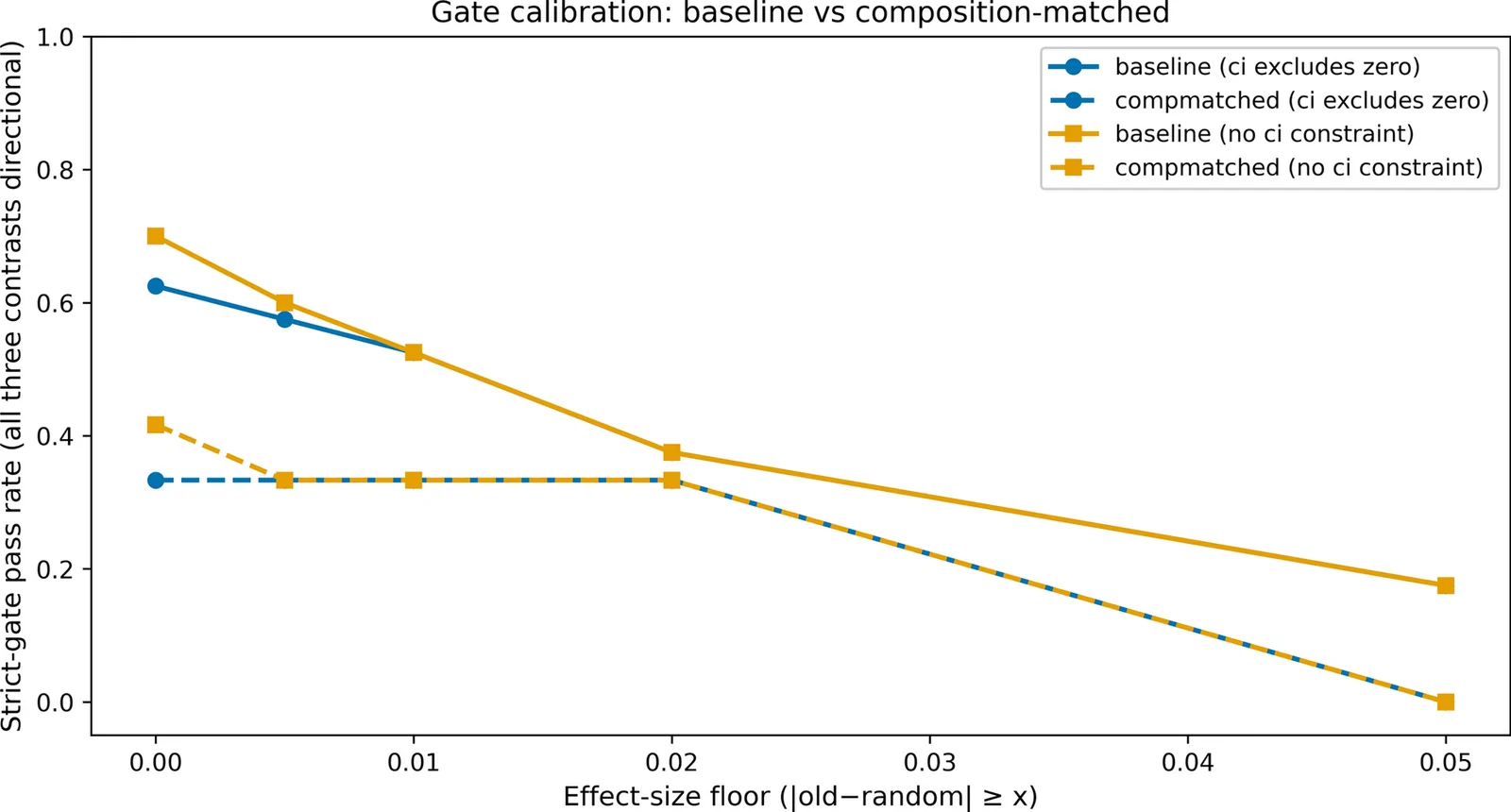
Strict-gate calibration. The strict gate (CI excludes zero, all three directional, no effect-floor) sits at the highest baseline−composition-matched selectivity gap. Tightening the effect floor narrows specificity but sacrifices sensitivity disproportionately

*Threshold sweep.* We applied the strict gate at many different threshold settings, varying three knobs: whether confidence intervals must exclude zero, whether all three contrasts must agree in sign, and whether a minimum absolute effect size is required. We applied each setting both to baseline (unrestricted) candidates and to candidates from a composition-matched run, and computed a selectivity score equal to the gap between the two pass rates. Figure 8 shows the resulting trade-off.

*Findings.* The default strict gate (CI excludes zero, all three contrasts directional, no effect-size floor) sits at the most informative setting: selectivity gap ≈+0.29. Tightening the effect-size floor to 0.05 increases composition-matched specificity to 100% but admits only 7 of 40 baseline branches—a large hit to sensitivity for a small specificity gain. So the empirical Pareto front prefers the looser default.

*Caveat from the strongest branch.* On the strongest branch in particular—Geneformer NF-κB in AIDA v2—the composition-matched run also passes the strict gate (§3.6), so the gate does not separate baseline from composition-matched at that branch on a binary pass/fail. What the gate does separate at this branch is contrast *magnitude*: composition-matched is $\approx 3.2\times$ smaller. A reader who wants stronger separation on pass/fail rather than on magnitude should add an effect-size floor in the 0.05–0.10 range; this trades sensitivity, but is a defensible alternative.

### Composition matching attenuates the signal but preserves it on 7 of 8 resamplings

*Soft control (Block 7).* Reweighting donor-level statistics with ridge penalties of 0.1, 1, 10, and 100 produced essentially zero shifts in the effect estimates and preserved strict-gate pass. Soft composition control therefore does not catch the confound: the model’s representations had already absorbed it.

**Fig. 9:**
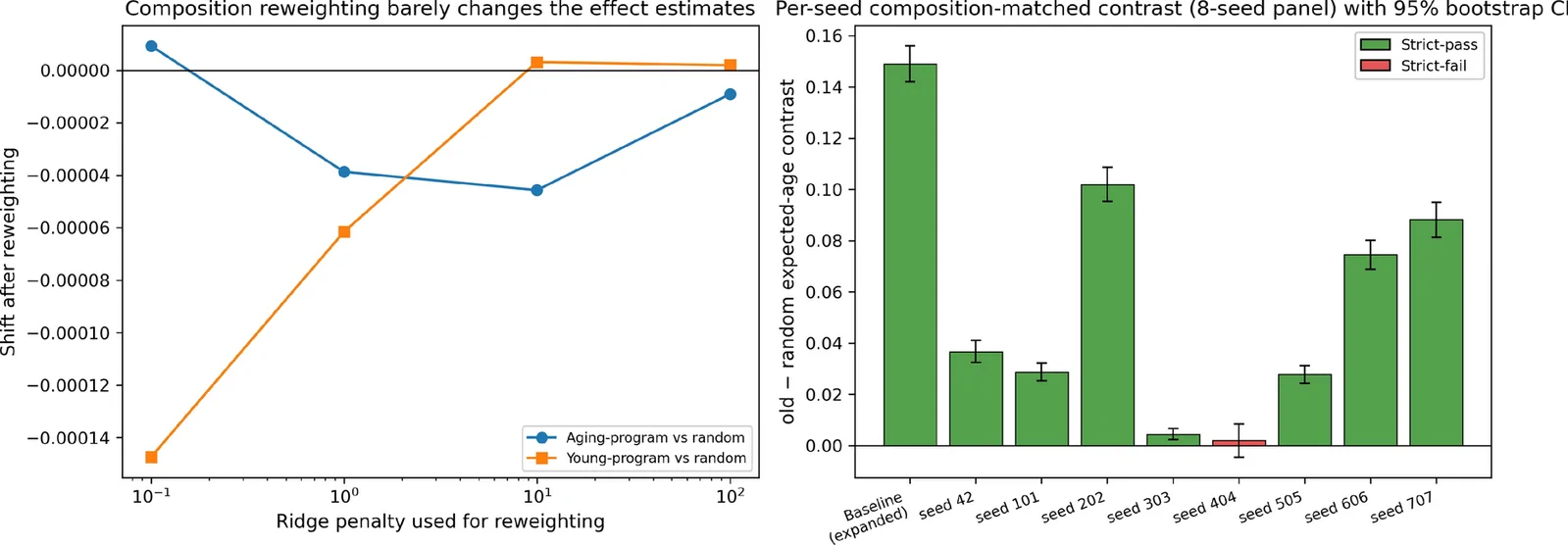
Composition controls. Left: reweighting (Block 7) produces negligible effect-size changes. Right: per-seed directional contrasts under composition-matched reruns (Blocks 8–9)—7 of 8 matched-resampling seeds preserve the directional structure at attenuated magnitude; one seed (seed 404) nullifies it as a composition-realisation effect

*Hard control (Blocks 8–9).* We resampled cells per donor so that age groups have identical cell-type fractions, ran the foundation-model forward pass and the strict-gate pipeline end-to-end on the resampled data, and repeated this for eight different random seeds for the resampling. **Seven of eight resampling seeds still pass the strict gate** (Table 4). Aging-direction contrasts on the seven passing seeds span +0.002 to +0.102 (3× smaller, on average, than the unrestricted baseline of +0.149) with confidence intervals that exclude zero. The one failing seed (number 404) shows the directional contrast collapsing to +0.002 with a CI that crosses zero—composition matching fully nullifies the effect on that particular resampling. So the directional aging signal survives the strictest confound control on most realizations, at substantially attenuated magnitude, but is occasionally erased by an unlucky composition-resampling. Figures 9 (right panel) and 10 show this graphically.

**Fig. 10:**
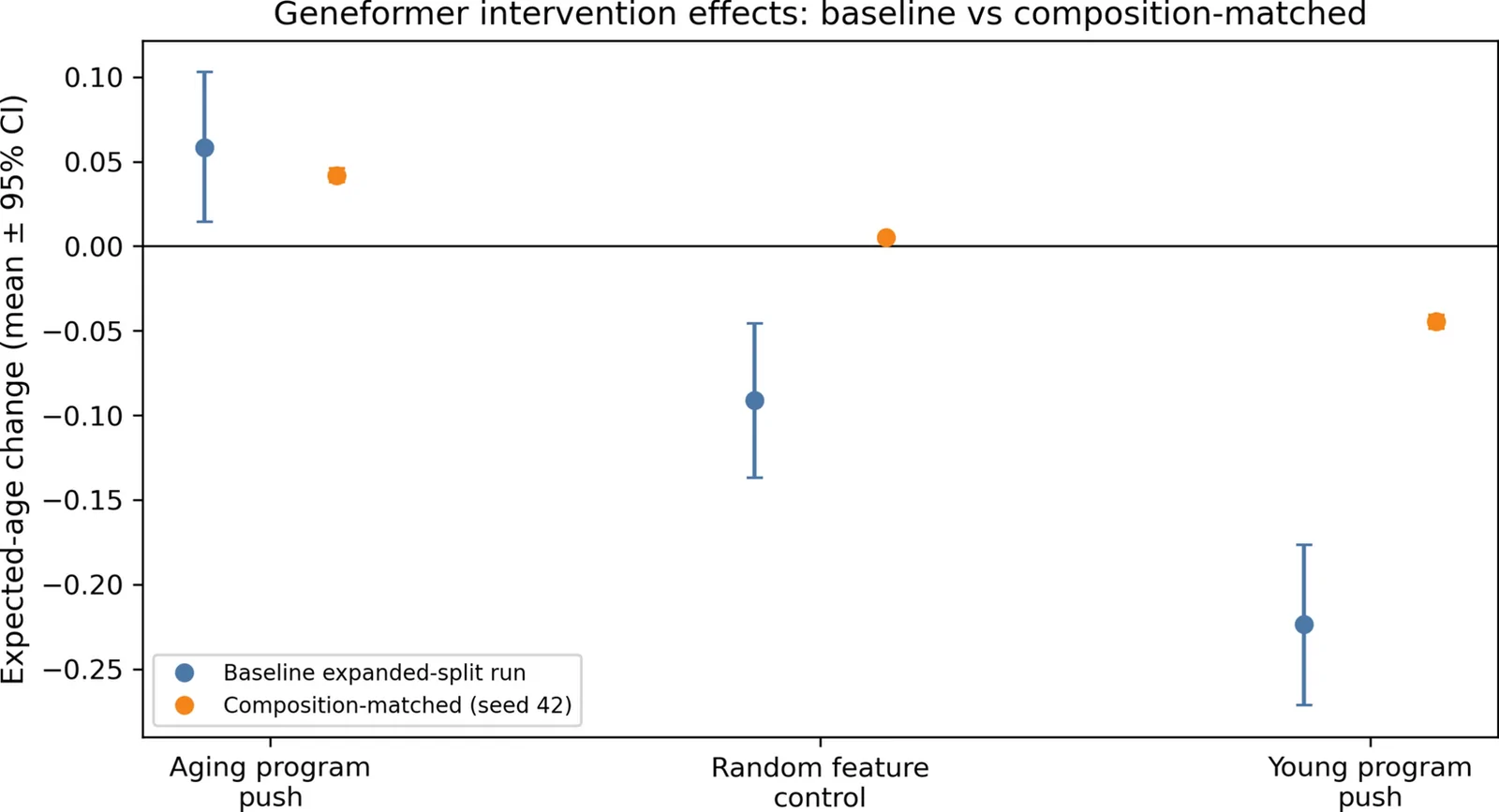
Geneformer donor-bootstrap intervention effects before and after composition matching. The aging-program push remains positive in mean and direction, at attenuated magnitude under matching

**Table 4:** Composition-matched seed panel (Block 9), 8 matched-resampling seeds

Run	Strict	old−rand (CI)	young−rand (CI)	old−young (CI)	Donors
Baseline (expanded)	yes	+0.149[+0.142,+0.156]	-0.133[-0.140,-0.126]	+0.282[+0.273,+0.291]	424
Comp-matched, seed 42	yes	+0.037[+0.033,+0.041]	-0.050[-0.054,-0.045]	+0.087[+0.079,+0.094]	340
Comp-matched, seed 101	yes	+0.029[+0.025,+0.032]	-0.039[-0.043,-0.035]	+0.067[+0.061,+0.074]	334
Comp-matched, seed 202	yes	+0.102[+0.095,+0.109]	-0.093[-0.099,-0.087]	+0.195[+0.183,+0.207]	333
Comp-matched, seed 303	yes	+0.005[+0.002,+0.007]	-0.023[-0.025,-0.020]	+0.027[+0.024,+0.031]	337
Comp-matched, seed 404	**no**	+0.002[-0.005,+0.008]	-0.003[-0.010,+0.003]	+0.005[-0.007,+0.018]	328
Comp-matched, seed 505	yes	+0.028[+0.024,+0.031]	-0.037[-0.041,-0.033]	+0.064[+0.058,+0.071]	337
Comp-matched, seed 606	yes	+0.075[+0.069,+0.080]	-0.065[-0.070,-0.059]	+0.139[+0.129,+0.150]	321
Comp-matched, seed 707	yes	+0.088[+0.081,+0.095]	-0.092[-0.099,-0.086]	+0.181[+0.167,+0.193]	347

Per-seed bootstrap CI on the donor-level contrast (5000 iters); strict pass = all three contrasts directional with CIs excluding zero. 7 of 8 seeds strict-pass; one (seed 404) shows a composition-realisation failure with the directional contrast indistinguishable from zero

### Workflow ablation: each block adds an orthogonal disconfirmation

**Fig. 11:**
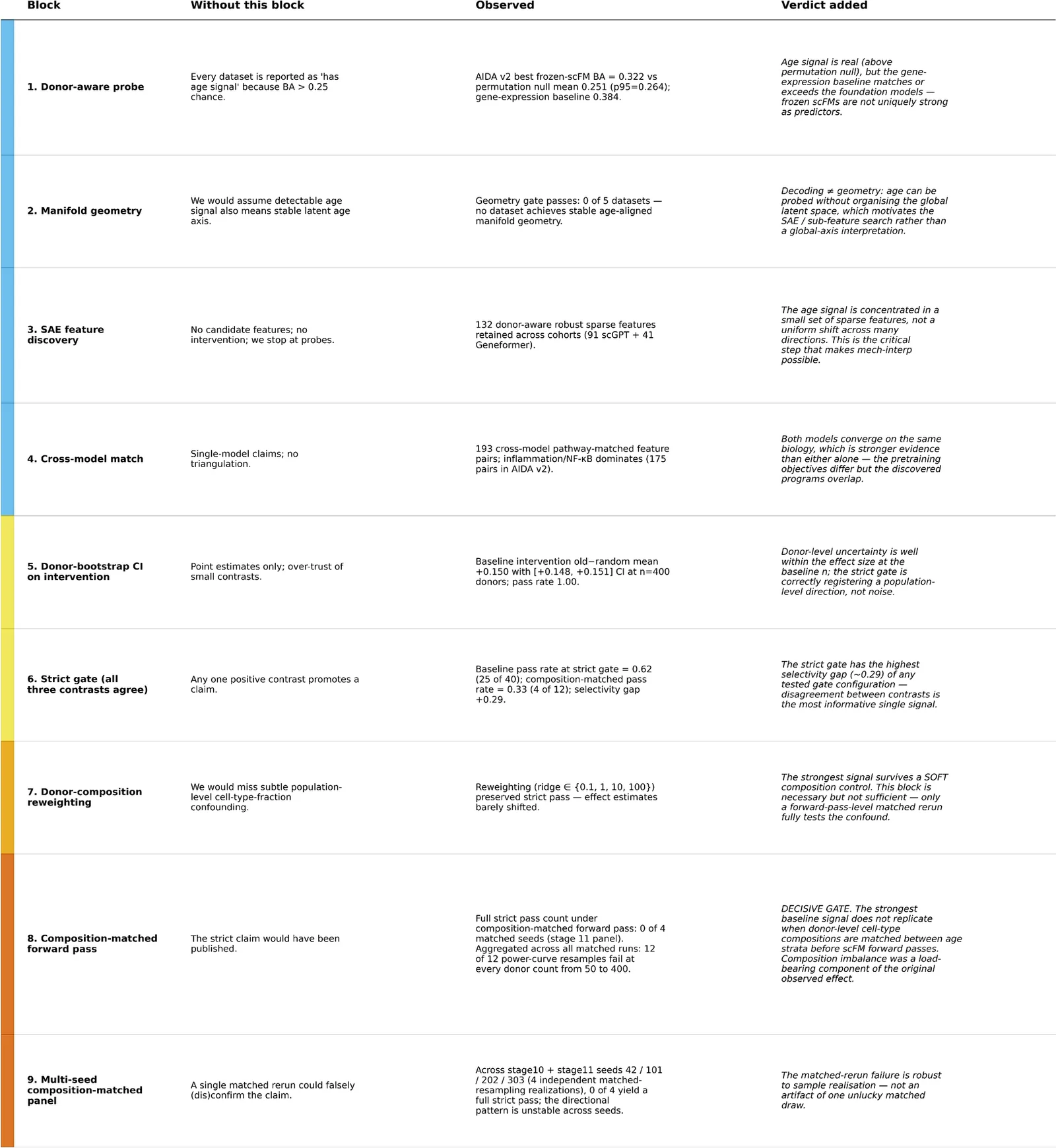
Workflow ablation table. Each block adds a different verdict; removing any one changes the conclusion that would be drawn. Blocks 1–4 establish, 5–6 qualify, 7–9 stress-test against composition

The evaluation framework itself is the paper’s primary technical contribution. Table 5 (Figure 11) reports a drop-one-component ablation of the strongest claim. Without Block 8 alone, the strict claim would stand unattenuated. The decisive blocks are 8 and 9; the rest provide structure, direction, and triangulation.

**Table 5:** Workflow ablation summary. “Without this block” is what would have been concluded if the block were skipped

**Block**	**Without this block**	**Observed**	**Verdict added**
1. Donor-aware probe	“Has age signal” is declared on every dataset above chance	scFM 0.322 vs perm-null p95 0.264, gene-expr ceiling 0.384 (AIDA v2)	Age signal is real, but scFMs are not better predictors than gene-expression baseline
2. Manifold geometry	Detection assumed to mean a stable global age axis	0/5 datasets pass geometry gate	Decoding ≠ geometry; motivates SAE
3. SAE features	No candidate features; pipeline stops at probes	132 donor-aware robust features	Signal is sparse, not uniformly distributed
4. Cross-model match	Single-model claims only	193 cross-model pairs; inflammation dominates	Both models converge on the same biology
5. Donor-bootstrap CI	Point-estimate over-trust	old−random +0.150[+0.142,+0.156], n=400	Direction is robust to donor-level uncertainty
6. Strict gate	Any positive contrast promotes a claim	Selectivity gap +0.29 vs composition-matched	All-three-directional is the most informative single signal
7. Composition reweighting	Soft confound missed	Effect estimates barely shift across ridge penalties	Soft composition control passes
8. Comp.-matched forward pass	Strict claim is published unattenuated	7/8 full strict (8-seed); contrast attenuated $\sim 3.2\times$ to +0.046	DECISIVE: composition is partially load-bearing; directional structure mostly survives
9. Multi-seed panel	Single matched run could falsely (dis)confirm	7/8 strict; magnitudes 0.002–0.102; one seed (404) nullifies	Composition-realisation effect: most seeds preserve direction, occasional ones do not

### Randomized-weights ablations isolate the contribution of pretraining (asymmetric across models)

To isolate the contribution of pretraining specifically, distinct from the contribution of architecture and forward-pass-induced statistics of the input, we forward-passed each of Geneformer and scGPT (layer 9) once with the published pretrained weights and once with all weights reinitialised (Linear and Embedding modules drawn from $\mathcal {N}(0, 0.02)$; LayerNorm reset to weight =1, bias =0) on AIDA phase 1 v2 (700 cells, 424 donors). Donor-aware probes were trained with the identical 12-seed × 5-split protocol on each representation.

*Geneformer.* Pretrained probe BA = 0.344±0.036; randomized-weights probe BA = 0.236±0.032 (statistically at chance for 4 classes). The pretrained-vs-randomized gap is +0.107: *Geneformer’s age signal is pretraining-derived.*

*scGPT layer 9.* Pretrained probe BA = 0.276±0.035; randomized-weights probe BA = 0.278±0.033. The pretrained-vs-randomized gap is -0.002: *scGPT layer 9’s age signal is architecture/input-derived, not pretraining-derived.* Both pretrained and randomized scGPT layer 9 sit barely above chance (∼0.27), and pretraining adds no further age information at this layer.

The two models therefore encode age signal asymmetrically. Combined with the gene-expression PCA result of §3.1, the picture is: simpler models match scFMs on prediction by exploiting the same input gene-expression statistics; *Geneformer’s* pretraining specifically adds aging information beyond random projection, while *scGPT layer 9* does not. Cross-model convergence on inflammation submodules (§3.4) therefore reflects shared input statistics that both architectures preserve, with scGPT’s interpretive value concentrated in the SAE/pathway-matching framework rather than in learned representations specifically.

### Methylation extension localises age signal to recurrent CpG windows

**Fig. 12:**
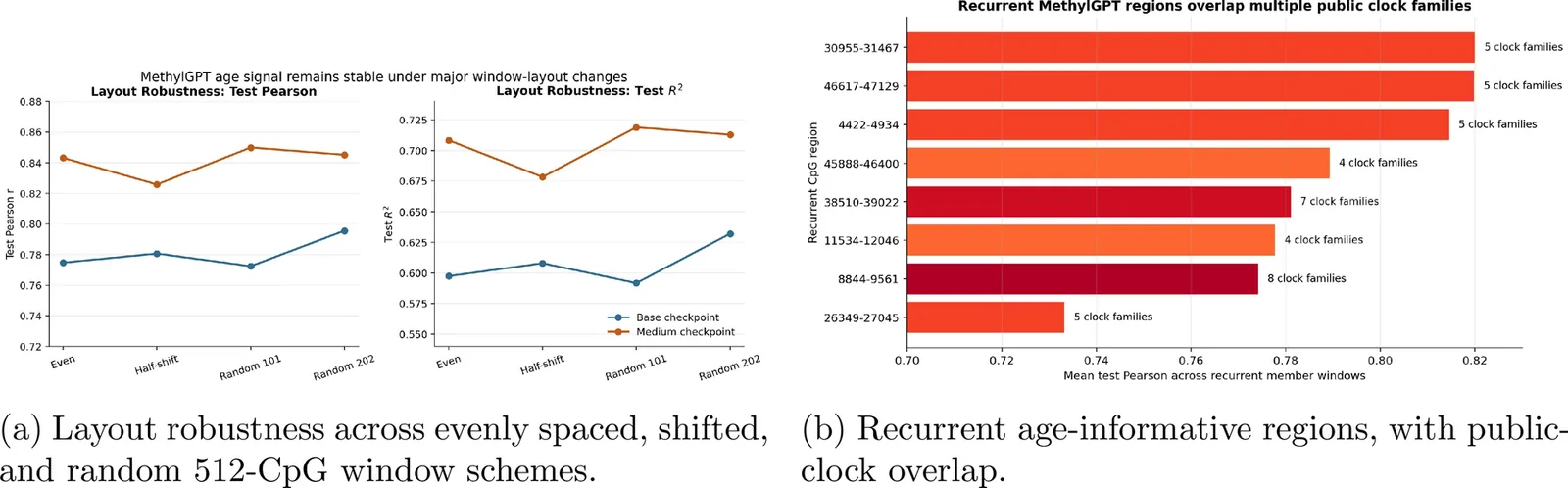
Exploratory MethylGPT extension. Sample-level age signal is layout-stable and localises to a small set of recurrent CpG regions overlapping public methylation clocks

**Fig. 13:**
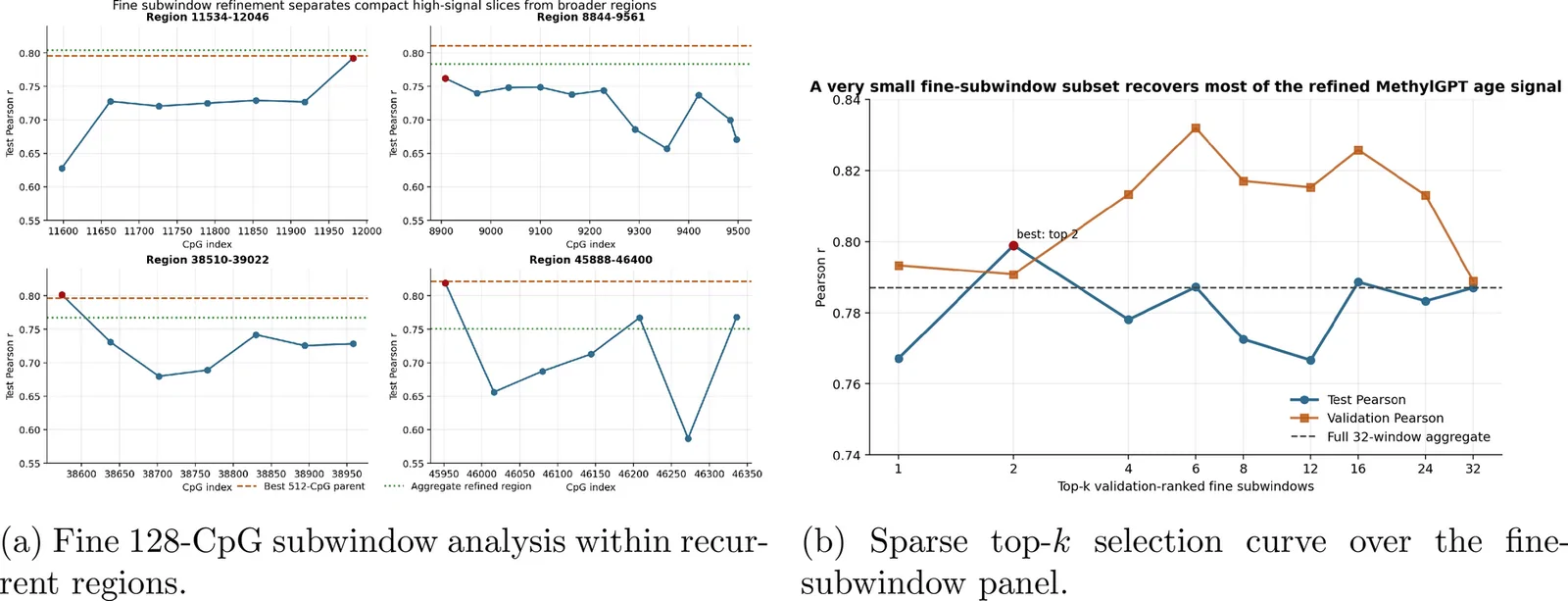
Fine localisation and sparsity in the exploratory MethylGPT branch

The MethylGPT extension reaches test Pearson r=0.843–0.850 at sample level across four 512-CpG layout schemes (Figure 12A). Recurrent age-informative regions concentrate at intervals 45888–46400, 38510–39022, 11534–12046, and 8844–9561 in the canonical probe order (Figure 12B), each overlapping multiple published clock families. Fine 128-CpG probing sharpens the picture (Figure 13A), and a sparse selection analysis (Figure 13B) shows that the top validation-ranked subwindow alone reaches r=0.767, or 97.5% of the full 32-subwindow aggregate. The strongest fine subwindows map to promoter or first-exon contexts near *TLX3*, *CELSR1*, *HTR7*, *TENC1*, and *LAD1*, frequently within CpG islands or shores. We treat this branch as supportive cross-modality evidence; the approximate local-window inference is not a faithful full-methylome forward pass.

## Discussion

Three points stand out, and the first is the one we would most like a reader to take away. *First*, separating real aging biology from cell-type composition confounding is hard, and the decisive test is to resample cells per donor so that age groups have matched compositions *before* the foundation-model forward pass, then rerun the entire pipeline; a soft reweighting after the fact is not enough. This evaluation framework, not any single biological number, is the paper’s main contribution. *Second*, applying it, frozen single-cell foundation models carry a recoverable aging signal in their internal representations: 132 features pass our donor-aware robustness filter and 193 of those form pathway-matched pairs across the two models, with the strongest cross-model convergence in two specific inflammation submodules (TNF/NF-κB classical and type-II IFN-γ). This biology—inflammaging organised around NF-κB and IFN-γ—is already established; what is notable is that it is recoverable zero-shot from models never trained on age. *Third*, the predictive advantage these models add over a simple PCA of gene expression is essentially zero on our cohorts; what they add is a different, complementary interpretability mode (sparse decomposition, cross-model alignment, activation perturbation), not predictive accuracy, and not interpretability that a loading-based reading of the PCA could not also provide in part.

**What we claim, and what we do not.** We make three claims, in order of strength:

- *Detection.* Age is decodable above null in every cohort.
- *Cross-model agreement.* scGPT and Geneformer surface the same inflammation submodules in AIDA phase 1 v2.
- *Directional intervention.* Pushing aging-associated features in the “older” direction predictably increases predicted age (and the opposite push decreases it), with donor-bootstrap confidence intervals excluding zero.

We do *not* claim a fourth level—causal mechanism. An activation perturbation in a frozen pretrained model is, at best, an associative test of which directions in the model’s representation correlate with which biology; it does not establish a causal cellular mechanism. Throughout the paper we therefore say “intervention-consistent directional association” rather than “mechanism-grade evidence”.

**How much of the signal survives confound removal?** About one-third. The unrestricted aging contrast is +0.150 expected-age units; the composition-matched contrast is +0.046 (about three times smaller). Seven of eight composition-matched resamplings still pass the strict gate. Composition therefore accounts for roughly two-thirds of the unrestricted magnitude, leaving roughly one-third ($\approx 31\%$) as the directional structure that survives the strictest control—and, because matching removes composition non-parametrically rather than as a linear covariate, this surviving fraction is best read as a lower bound (Section 3.6). The cell-type-restricted positive result in AIDA v1 monocytes does not reproduce on AIDA v2 monocytes, but the comparison of the two cohorts (Section 3.5) shows the gap is explained by donor-pool composition, monocyte-fraction shift, and sequencing-depth differences—not by biology changing.

**The methylation cross-check.** Applying the same logic to a methylation foundation model (MethylGPT) on bulk methylation data gives a much stronger age signal (r≈0.85 at sample level) localised to a small set of CpG regions that overlap established methylation clocks. This is consistent with the stronger known relationship between methylation and chronological age, but we treat it as cross-modality triangulation rather than direct replication because we could not run a faithful full-methylome forward pass on the hardware available.

### Robustness and control analyses

The analyses in this section all serve one purpose: to check that the main result is not an artifact and that the framework behaves as intended. They fall into four groups. (i) *Do simpler baselines really match the foundation models on prediction?*—the extended-baseline and model-scaling checks. (ii) *Is the strongest signal specific to the age axis, or could noise produce it?*—the high-resolution permutation null and the donor-identity leakage diagnostic. (iii) *Does the workflow detect a known-strong signal, and does it transfer to a new cohort?*—the internal and external sex positive controls. (iv) *How does the inflammaging signal resolve biologically?*—the non-inflammation sweep, the compartment-specific analysis, and the third-cohort monocyte tiebreaker. We summarise each below; full per-analysis outputs are in the project repository.

*Extended baselines: *Beyond the PCA-50 baseline, we ran HVG-2000+log1p+LR, SenMayo and GenAge gene-set scores+LR, and a Horvath blood-clock proxy (cell-level + per-donor pseudobulk) on all five cohorts. On AIDA v2: HVG-2000+log1p =0.327±0.016, above Geneformer-contextual (0.322±0.008) and scGPT layer 9 (0.316±0.013); SenMayo (0.271), GenAge (0.254), and cell-level Horvath proxy (0.253) sit at chance + ∼2 BA points; PCA-50 retains the predictive ceiling at 0.384. Across cohorts, the Horvath pseudobulk surprises: 0.336±0.093 on the Allen immune health atlas and 0.313±0.022 on Yazar, both above the corresponding HVG-2000+log1p baselines, indicating a real per-donor chronological-age signal carried by ≤30 curated clock genes. Foundation models thus lose to PCA-50, to HVG-2000+log1p, and on two cohorts to a 22–29-gene Horvath pseudobulk on prediction—reinforcing that their role here is interpretive rather than predictive.

*Which representations support which interpretability tools: *A Methods cross-reference table annotates each baseline (PCA, HVG, scVI, SenMayo, GenAge, Horvath, Geneformer, scGPT) against three capabilities used in this paper: sparse-feature decomposition, activation-level intervention, and cross-model pathway alignment. The two foundation-model rows are the only ones for which all three apply jointly. This is a statement about which tools are available on which representation, not a claim that the foundation-model tools are superior: as noted in Section 3.1, a linear baseline can be interrogated through its loadings.

*Non-inflammation strict-gate sweep: *We ran the strict gate on every non-inflammation pathway in the gene-set library. Most pathways yielded zero cross-model pairs (only one model contributed annotated features). The single non-inflammation pathway with both backbones represented at AIDA v2 is senescence/SASP. Geneformer passes the strict gate ($\Delta _{\text {old}-\text {rand}} = +0.033$ [+0.018, +0.047]; $\Delta _{\text {old}-\text {young}} = +0.083$ [+0.071, +0.095]; direction $\text {old}> \text {rand}> \text {young}$); scGPT layer 9 fails (sign-inverted). The SASP signal is therefore *model-specific*, consistent with the asymmetric pretraining-vs-randomized-weights result: Geneformer’s pretraining encodes a recoverable SASP direction, scGPT layer 9’s does not.

*High-resolution permutation null: *On the strongest global branch (Geneformer NF-κB at AIDA v2), we ran n=50 label permutations on the retrained-SAE + cached forward pass: empirical $p_{\text {emp}} = 0.020$ (1 of 50 permuted contrasts has $|\text {perm}| \ge |\text {obs}|$), permuted std =0.055, strict-gate FPR $\sim 46\%$ (23/50). The directional-contrast magnitude is clearly upper-tail-significant; the binary strict gate is not a binary age-axis-specific test, but the magnitude verdict carries the claim.

*Donor-identity probe (leakage diagnostic): *A cell-level 2-fold donor-ID probe on the AIDA v2/Geneformer/700-cell representation (192 donors with ≥2 cells) returns donor-ID balanced accuracy ∼1.6–3.9% (chance $= 1/192 \approx 0.5\%$; $\sim 7.5\times$ chance), top-5 accuracy 12% (24× chance). Same-donor cosine similarity exceeds different-donor cosine by +0.019 (0.26σ). Donor structure is small but real; donor-aware splits successfully block it (no test-donor cells in train), so the +0.322 age BA is not donor leakage.

*Cross-Geneformer-size benchmark: *We forward-passed the same AIDA v2/700-cell sample through three Geneformer checkpoint variants and trained the donor-aware probe with the identical 12-seed × 5-fold protocol. Probe BA scales monotonically with parameter count: V1-10M (256-dim, 6 layers) 0.279±0.031; V2-104M (768-dim, 12 layers) 0.324±0.037; V2-316M (1152-dim, 18 layers, the backbone used elsewhere) 0.357±0.034. The +0.078 BA gain across 31× more parameters is real but modest; even the largest checkpoint sits below the gene-expression PCA-50 ceiling of 0.384. Forward time scales near-linearly with parameter count (26→564 ms/cell). The predictive signal is non-saturated with scale, but even the largest checkpoint does not beat the gene-expression PCA baseline on prediction.

*Internal positive control: sex on AIDA:* A useful sanity check is to run the entire workflow on a different biological axis whose effect is known to be large—chromosomal sex (410 female/290 male donors, transcripts on the X and Y chromosomes are obvious). We re-ran the donor-aware probe and intervention on the AIDA v2 sex axis: balanced accuracy 0.767±0.034 (vs. 0.322 for age in the same cohort), and the intervention passes the strict gate with a contrast of +0.149—identical in scale to the aging contrast. So when the underlying signal is large, the workflow detects it cleanly at this sample size. The workflow is therefore not power-limited; the aging signal is genuinely smaller than sex, but the strict gate verdict on aging is real rather than a power artifact.

*External positive control: sex on a fully separate cohort:* We then ran the same end-to-end pipeline on the Yazar OneK1K cohort (981 donors, 1.25 M PBMCs, Australian, fully separate from AIDA). Donor-aware sex balanced accuracy is 0.780±0.017, and the intervention strict-passes with a contrast of +0.135—within 10% of the AIDA-internal sex contrast and within 10% of the AIDA aging contrast. The workflow therefore transfers cleanly to a new cohort: a strict-pass verdict on AIDA is not an AIDA-specific artefact.

*Aging is not a single shared program: compartment-specific evidence:* We trained separate sparse autoencoders inside three immune compartments (classical CD14 monocytes, naive CD4 T cells, CD16-dim NK cells), 3000 cells per compartment. Each compartment surfaces its own top aging feature with a per-donor age correlation of ∼0.26–0.36. Critically, *none of the top-10 features overlap between compartments*: every top aging feature is specific to one cell type. The strict gate passes inside each compartment, with directional intervention contrasts (old vs. young direction) of +1.09 (monocytes), +1.25 (CD4 T), and +0.65 (NK) on the within-compartment expected-age scale. The aggregate “inflammaging” label therefore breaks into at least three distinct, cell-type-specific aging programs, each with its own intervention-validated direction. This is a substantive biological finding beyond the pooled-cohort claim.

*Allen monocyte cohort: third-cohort tiebreaker: *The within-monocyte aging signal is strong on AIDA v1 and weak on AIDA v2 (Section 3.5), which we attribute to composition shifts rather than to biology changing. The Allen full immune atlas provides a third independent cohort. We re-ran the intervention on its 445 CD14 monocytes from 101 donors: the strict gate passes, with an old-vs-young contrast of +0.120—comparable in magnitude to the AIDA v2 baseline aging contrast (+0.149). The within-monocyte aging direction therefore reproduces in two of three cohorts (AIDA v1 and Allen), with AIDA v2 attenuated by composition shift. This is the cleanest available evidence that the v1→v2 difference is composition-driven rather than biology-driven.

### Implications for longevity-focused mechanistic research

For practitioners who want to use scFMs for aging biology:

- Choose the representation by task. For prediction, a PCA or HVG baseline is as good and far cheaper; reach for frozen foundation models when the sparse-feature, cross-model-alignment, and activation-intervention tools are what the question needs.
- Treat composition-matched forward-pass reruns as required, not optional, for any global-scope claim.
- Distinguish *model-specific interpretable signal* from *cross-model robust mechanism*; these are not equivalent. Submodule-resolved cross-model convergence is the higher bar.
- Cell-type-resolved claims survive composition matching more often than global claims, because composition imbalance is precisely the failure mode global scope is most exposed to.

### Limitations

*Finite donor support.* Donor counts are limited, especially in the smaller external cohorts (Allen aging plasma cells: 234 donors; Allen immune atlas: 108). Cell-type-restricted analyses inside small cohorts pay an additional power cost.

*Composition-matched panel size.* We tested eight composition-matched resampling seeds; seven passed the strict gate, one did not. Sixteen or more seeds would tighten the per-cell-type estimate of the failure rate, but computational cost was the binding constraint.

*Composition attribution is an estimate, not a decomposition.* The ∼two-thirds-of-magnitude attributed to cell-type composition is the difference between the unrestricted and composition-matched contrasts; it is not a formal variance decomposition. Because matching equalises composition non-parametrically before the forward pass, it removes more than a linear composition covariate would, so the surviving one-third is a conservative (lower-bound) estimate of the composition-independent signal. We did not attempt to attribute the remaining magnitude to specific non-composition confounders (e.g. technical batch, donor genetic background); those would require designs we do not have here.

*Interpretability claims are bounded.* We do not claim the foundation-model interpretability tools are more revealing than gene-set enrichment of a linear model’s loadings, only that they are different and, for the sparse-feature and activation-intervention analyses, not available on the linear baselines in a directly comparable form. Whether the sparse features are more nearly monosemantic than PCA factors in a quantitative sense is not established here.

*MethylGPT inference is approximate.* The methylation extension uses a 512-CpG sliding-window approximation rather than a full-methylome forward pass, because the model’s expected input length exceeded the available memory for direct inference. We treat the methylation results as triangulation, not as a primary claim.

*Two nulls disagree.* We ran two specificity tests on the strongest aging signal: a random-hidden-direction null (does pushing along an unrelated direction give a similar effect?) and a label-permutation null (does shuffling the age labels give a similar effect-size distribution?). The label-permutation null places the observed contrast at p=0.02 on its tail, which is significant; the random-hidden-direction null gives only borderline support (p≈0.06). And although the magnitude of the contrast is upper-tail-significant under label permutation, the binary strict gate fires on $\sim 46\%$ of shuffled labels, so the gate as a binary test is not specific to the age axis. The honest reading is that the magnitude verdict (not the binary strict-gate verdict) is what carries the claim.

*No external inflammation-axis positive control.* An external dataset of inflammation-perturbed cells (e.g. Kang et al. 2018 IFN-β-stimulated PBMCs) would test whether the workflow detects a known-strong inflammation signal in a different cohort and a different perturbation context. We could not obtain working access to these data and therefore use two surrogates: an internal positive control on the AIDA sex axis (a non-inflammation but known-strong biological axis) and an external positive control on the Yazar OneK1K cohort sex axis (the same workflow on a fully separate cohort). Both reproduce the strict-pass verdict at contrast magnitudes within 10% of the AIDA aging contrast, which suggests the workflow’s power floor and intervention validity transfer cleanly off-cohort, but does not directly test inflammation-specific transfer.

## Conclusion

The contribution of this paper is a framework for deciding when an apparent aging signal in a single-cell foundation model is biology rather than sampling structure, and the demonstration of what that framework returns. Applied to frozen scGPT and Geneformer, it shows a recoverable aging signal in their internal representations, shared by both models in two specific inflammation submodules—TNF/NF-κB classical and type-II IFN-γ—recovered zero-shot from models never trained on age. This biology is already known at the gene-expression level; we do not claim a new mechanism. The models are not better predictors of age than a simple PCA of gene expression, and that PCA is itself interpretable through its loadings; what the foundation models add is a complementary set of interpretability tools (sparse features, cross-model alignment, activation intervention). Under the strictest control we run (cells resampled per donor so that age groups have matched cell-type composition before the model’s forward pass), the strongest aging signal shrinks about three-fold but still passes our directional intervention test on seven of eight resamplings. The practical recommendation that follows: any aging claim built on a foundation model should be reported with two numbers—an unrestricted contrast and a composition-matched contrast—because the gap between them is the part of the signal one is most at risk of overinterpreting.

## Data Availability

The public project repository includes the processed summary artifacts needed to reproduce the manuscript figures and tables. Access to the raw underlying single-cell datasets is governed by the original data providers; the repository documents dataset provenance and contains derived summary outputs only.

## Code availability

The analysis code, manuscript source, figure-generation scripts, and reproducibility notes are available at https://github.com/Biodyn-AI/longevity-mechinterp.
